# The 150 most important questions in cancer research and clinical oncology series: Questions 25–30: Edited by *Chinese Journal of Cancer*

**DOI:** 10.1186/s40880-017-0210-y

**Published:** 2017-05-04

**Authors:** Chinese Journal of Cancer

**Affiliations:** Editorial Office of Chinese Journal of Cancer, Sun Yat-sen University Cancer Center, Guangzhou, 510060 Guangdong P. R. China

**Keywords:** Immune responses, Immunotherapy, Metastasis, Cancer initiation

## Abstract

To accelerate our endeavors to overcome cancer, *Chinese Journal of Cancer* has launched a program of publishing 150 most important questions in cancer research and clinical oncology. In this article, 6 more questions are presented as followed. Question 25: Does imprinting of immune responses to infections early in life predict future risk of childhood and adult cancers? Question 26: How to induce homogeneous tumor antigen expression in a heterogeneous tumor mass to enhance the efficacy of cancer immunotherapy? Question 27: Could we enhance the therapeutic effects of immunotherapy by targeting multiple tumor antigens simultaneously or sequentially? Question 28: Can immuno-targeting to cytokines halt cancer metastasis? Question 29: How can we dynamically and less-invasively monitor the activity of CD8^+^ T killer cells at tumor sites and draining lymph nodes? Question 30: How can the immune system destroy the niches for cancer initiation?

To accelerate our endeavors to overcome cancer, *Chinese Journal of Cancer* has launched a program of publishing 150 most important questions in cancer research and clinical oncology [1], with the first 24 questions been published in the last four issues [2–5]. In this article, questions 25–30 are selected and presented. *Chinese Journal of Cancer* is still open to collect more key questions in cancer research and clinical oncology. Please send us your thoughtful questions to Ms. Ji Ruan via email: ruanji@sysucc.org.cn.

## Question 25: Does imprinting of immune responses to infections early in life predict future risk of childhood and adult cancers?

### Background and implications

Innate and adaptive immune responses to pathogens are known to vary by age at initial exposure. A classical example is the different responses observed to first exposure to Epstein–Barr virus (EBV) in early life (mild immune response; asymptomatic) versus adolescence (vigorous response often leading to infectious mononucleosis). It has long been hypothesized that differential immune responses established in early life influence risk of various cancers, including lymphomas (e.g., Hodgkin’s lymphoma, HL) and some solid tumors (e.g., nasopharyngeal carcinoma, NPC). In fact, evidence suggests that individuals initially exposed to the ubiquitous EBV during adolescence (as indicated by a diagnosis of mononucleosis) have a high risk of subsequent HL presumably due to lymphocyte hyperproliferation/expansion in response to late EBV exposure. In contrast, individuals initially exposed to EBV during adolescence appear to have a reduced risk of NPC, another EBV-associated cancer, and this is believed to be due to the development of more robust mechanisms for immune control of latent EBV infection (i.e., ability to limit chronic viral reactivation from latency) when infection first occurs late in life.

Within an early-life cohort, it would be possible to incorporate (1) a collection of biospecimens to permit assessment of exposure to specific pathogens, innate/adaptive immune responses to these pathogens, and more generalized markers of immune response, (2) a more actively followed subcohort within the larger early-life cohort in which timing of initial infections with specific pathogens and the immune responses to these pathogens can be evaluated in more detail, and (3) the use of the results from the study of this subcohort to inform findings observed in the larger early-life cohort. Childhood and young adult cancers could serve as the primary outcome in the larger cohort because they usually occur within a reasonable number of years after cohort enrollment. Adult-onset cancers would take too long to evaluate directly within the early-life cohort because they would not occur for many decades after cohort enrollment. For these cancers, the evaluation of well-established immunological markers (called intermediates) of cancer risk (e.g., IgA-rich anti-EBV antibody responses or chronic EBV reactivation/shedding) would serve as the primary outcome in the larger cohort.

Note: The above examples focus on EBV and its associated cancers because this is my personal area of expertise, but the approach is broadly applicable to other cancers known or hypothesized to be associated with infections and/or chronic immune stimulation.

#### Submitter

Allan Hildesheim.

#### Affiliation and email

National Cancer Institute, Bethesda, MD, USA.

hildesha@mail.nih.gov

## Question 26: How to induce homogeneous tumor antigen expression in a heterogeneous tumor mass to enhance the efficacy of cancer immunotherapy?

### Background and implications

Despite the impressive therapeutic effect on some types of cancer, immunotherapy-induced anti-tumor immune response is often not potent enough to kill all cancer cells. Cancer immunotherapy induces immunity against cancer cells expressing tumor antigens that can be recognized by the immune system. Intra-tumor heterogeneity precludes expression of the same tumor antigens on all tumor cells, thereby causing immune evasion and therapy failure.

Can we induce a homogenous tumor antigen expression in the heterogeneous tumor mass, so as to expand the antitumor immunity and enhance the immunotherapy efficacy? Pre-treating the tumor with chemotherapy or radiation therapy, or even bacteria or virus vaccine, to induce a more homogeneous tumor antigen expression in the tumor mass may be able to enhance the antigen-specific antitumor immunity that will target the whole tumor mass and achieve better efficacy.

#### Submitter

Xiaojun Xia.

#### Affiliation and email

Sun Yat-sen University Cancer Center, Guangzhou, Guangdong, P. R. China.

xiaxj@sysucc.org.cn

## Question 27: Could we enhance the therapeutic effects of immunotherapy by targeting multiple tumor antigens simultaneously or sequentially?

### Background and implications

Cancer immunotherapy relies on the immune system to recognize and kill tumor cells expressing tumor antigens. Targeting one specific tumor antigen such as CD19 has been proved to be very successful for chimeric antigen receptor T cell (CAR-T) immunotherapy against B-cell-originated chronic lymphacytic leukemia. However, the tumor usually comes back at one year or longer after CAR-T immunotherapy. In addition, most solid cancer tissues are more heterogeneous than hematological malignancies, harboring dynamic and accumulating mutations, thus targeting a single tumor antigen is not sufficient for treating cancer. Given the rapidly advancing sequencing technology, we can now afford to quickly identify potential tumor antigens from individual patients. However, it remains unclear whether we could enhance the therapeutic effects of immunotherapy by targeting multiple tumor antigens simultaneously or sequentially. The exploration on targeting multiple tumor antigens simultaneously or sequentially would hopefully significantly enhance immunotherapy efficacy.

#### Submitter

Xiaojun Xia.

#### Affiliation and email

Sun Yat-sen University Cancer Center, Guangzhou, Guangdong, P. R. China.

xiaxj@sysucc.org.cn

## Question 28: Can immuno-targeting to cytokines halt cancer metastasis?

### Background and implications

Fighting cancer with immunotherapy is currently a promising direction in cancer research. Cancer immunotherapy possesses the advantages of tumor-specificity, immuno-adaptation, and relatively durable response. Our previous study has clearly demonstrated that Serglycin plays an instrumental role in driving metastasis of nasopharyngeal carcinoma. The following report from others also shows that Serglycin is critical in breast cancer metastasis. In addition, a previous study supports that Serglycin is the ligand of a cell surface protein CD44, which is an important marker in cancer stem cells. Serglycin is a highly glycosylated cytokine (the core protein is only 18 kDa, but its molecular weight increases to 300 kDa after fully glycosylation); it promotes cell motility by facilitating a variety of cytokines binding to cancer cell surface. Immunotherapy is applicable to targeting large proteoglycans such as Serglycin due to their characteristics of secretion. Natural killer cells and T cells activated by the immunogenicity of Serglycin would be perfect scavengers of secreted Serglycin. It is believed that developing novel therapeutic strategies by increasing the immunogenicity of Serglycin, for example conjugating Serglycin to a large carrier molecule, will lead to immuno-cleanup of Serglycin and subsequent attenuation of cancer metastasis.

#### Submitter

Xinjian Li.

#### Affiliation and email

The University of Texas MD Anderson Cancer Center, Houston, TX, USA.

xli13@mdanderson.org

## Question 29: How can we dynamically and less-invasively monitor the activity of CD8^+^ T killer cells at tumor sites and draining lymph nodes?

### Background and implications

With the approval of checkpoint inhibitors and many immunotherapeutics in development, personalized medicine approach is urgently needed to better select patients who have the best chance to respond to these expensive medicines so as to better control healthcare expenses and avoid unnecessary immune toxicities. CD8^+^ T cells play a crucial role in tumor cell killing mediated by these immunotherapeutics. However, it is impractical to get serial biopsies in patients to assess the dynamic status of these cells. A non- or less-invasive imaging approach may be able to fulfill this need, and will have the potential to transform clinical development and clinical use of this new class of cancer therapies.

#### Submitter

Li Yan.

#### Affiliation and email

GlaxoSmithKline, Brentford, London, UK.

li.1.yan@gsk.com

## Question 30: How can the immune system destroy the niches for cancer initiation?

### Background and implications

Basic immune response to cancerous cells in humans is the immune elimination of these cells. However, in cancer patients this does not work effectively. Alternatively, it may be hypothetical that, in individuals apparently without cancer, this immune surveillance remains perfect and it successfully eliminates initial cancers from the host and thereby rendering the host free of cancer for a lifetime, considering that the genetic and molecular changes would lead to persistently altered function in the individual. Whether cancer is resulted from the viral infection of stem cell niches in various tissues is of concern. Cancer stem cell niches are protected from immune recognition by cells within such niches or “niche guardians” such as pericytes, integrins, and laminans. Thus, an injured tissue could signal to a niche for recruitment of stem cells for tissue repair. In addition, the mutated cells could move from the viral compromised niche to the injured tissue and differentiate. When they do not succeed in repairing the wound, the tissue would signal for more repairs from the same niche, inducing a chain reaction of recruitment to the site and resulting in a growth of mutated cells. These cells are genomically varied because they are differentiated from the mutated stem cells. This process thus leads to cancer in the tissue or organ concerned. The host immunity fails to recognize the compromised niches. The research objective should be to identify underlying host immune activation mechanisms that will trigger the immune system to work against such “niches” and lead to probable cure at the root of cancer.

#### Submitter

Manigreeva Krishnatreya.

#### Affiliation and email

Dr. Bhubaneswar Borooah Cancer Institute, Guwahati, India.

Email: manigreeva@gmail.com; manigreeva@bbci.in
